# Comparative Study Assessing the Canal Cleanliness Using Automated Device and Conventional Syringe Needle for Root Canal Irrigation—An Ex-Vivo Study

**DOI:** 10.3390/ma15186184

**Published:** 2022-09-06

**Authors:** Keerthika Rajamanickam, Kavalipurapu Venkata Teja, Sindhu Ramesh, Abdulaziz S. AbuMelha, Mazen F. Alkahtany, Khalid H. Almadi, Sarah Ahmed Bahammam, Krishnamachari Janani, Sahil Choudhari, Jerry Jose, Kumar Chandan Srivastava, Deepti Shrivastava, Shankargouda Patil

**Affiliations:** 1 Department of Conservative Dentistry and Endodontics, Saveetha Dental College and Hospitals, Saveetha Institute of Medical and Technical Sciences, Chennai 600077, Tamil Nadu, India; 2 Restorative Dental Science Department, College of Dentistry, King Khalid University, Abha 62529, Saudi Arabia; 3 Department of RDS, Division of Endodontics, College of Dentistry, King Saud University, Riyadh 11545, Saudi Arabia; 4 Department of Pediatric Dentistry and Orthodontics, College of Dentistry, Taibah University, P.O. Box 344, Medina 42353, Saudi Arabia; 5 Department of Conservative Dentistry and Endodontics, SRM Dental College, SRM Institute of Science & Technology, Chennai 600089, Tamil Nadu, India; 6 Private Practice, Aluva, Ernakulam District, Kochi 683106, Kerala, India; 7 Department of Oral & Maxillofacial Surgery & Diagnostic Sciences, College of Dentistry, Jouf University, Sakaka 72388, Saudi Arabia; 8 Department of Preventive Dentistry, College of Dentistry, Jouf University, Sakaka 72388, Saudi Arabia; 9 Department of Maxillofacial Surgery and Diagnostic Sciences, Division of Oral Pathology, College of Dentistry, Jazan University, Jazan 45142, Saudi Arabia; 10 Centre of Molecular Medicine and Diagnostics (COMManD), Saveetha Dental College & Hospitals, Saveetha Institute of Medical and Technical Sciences, Saveetha University, Chennai 600077, Tamil Nadu, India

**Keywords:** endodontic materials, automated root canal irrigation, manual syringe needle irrigation, root canal treatment, smear layer

## Abstract

The success of endodontic treatment relies on both apical and coronal sealing. To achieve a good three-dimensional seal, the removal of the smear layer becomes mandatory. This study aims to assess the difference in debris accumulation and smear layer formation while using automated root canal irrigation and conventional syringe needle irrigation. Single-rooted human mandibular premolar teeth (*n* = 30) which were indicated for orthodontic extractions were selected. An endodontic access cavity was prepared, and a glide path was created. Based on the irrigation protocol decided upon for the study, the teeth were randomly allocated into three study groups, namely Group 1, where the manual syringe needle irrigation method was adopted; Group 2, in which automated root canal irrigation was undertaken; and Group 3, in which teeth remained un-instrumented as it was considered the Control group. The teeth were decoronated at the cement-enamel junction (CEJ) and were subjected for scanning electron microscopy (SEM) examination. Debris and smear layers were viewed in 1000× magnification and scored. A statistically significant (*p* < 0.05) lower mean debris and smear layer score (*p* < 0.05) was observed in both study groups when compared with the control group. However, no significant difference (*p* > 0.05) in the debris and smear layer was observed between the manual syringe needle irrigation and automated irrigation, although automated irrigation devices can be a potential alternative. The present study concluded that the efficacy of smear layer removal remained the same with both automated irrigation and manual syringe irrigation.

## 1. Introduction

Biomechanical preparation is a crucial intermediate phase in root canal therapy, as it helps the irrigant to thoroughly disinfect the root canal system [1,2]. Evidence states that root canal preparation forms a smear layer which covers the root canal walls randomly up to 2–5 μm thickness [3,4]. Primarily, the smear layer is a crystalline structure containing remnants of pulp, dentinal debris, microorganisms, and their products [5]. It is usually contaminated and retains bacteria in the dentinal tubules, thereby limiting the optimal penetration of disinfecting agents such as irrigants and intracanal medicaments [6,7,8]. The smear layer contributes to increased coronal and apical microleakage by interfering with the penetration of root canal sealer [9,10]. Therefore, eliminating the tenacious smear layer is essential in order to achieve adequate disinfection [6] and to enhance the fluid-tight closure of the root canal system [11].

The preparation of the apical one-third segment of the root canal is a challenging task as the canals here are more constricted and curved with ramification [12]. Studies have proven that smear formation is more significant at the apical one third of the root canal, and it is quite challenging to clean and disinfect, due to the inherent anatomical complexities [13]. A recent study demonstrated that irrespective of the technique employed and the subjection to various irrigant agitation techniques, there is a formation of the smear layer at the apical one-third [14]. Hence the apical one third is the most critical and difficult part of the root canal to shape and clean [13,15,16]. Only a small number of authors believe that clearly laid clinical research is required to completely comprehend the effects of eliminating the smear layer and treatment outcomes. According to the data from a systematic review, the results of root canal therapy seem to be improved with the elimination of the smear layer, [4,6]. The information that is now available therefore supports eliminating of the smear layer before proceeding with root canal obturation [17].

Previous studies have discussed the importance of chemo-mechanical debridement methods in removing the adherent inorganic and organic smear layer from the root canal system [18,19]. The use of sodium hypochlorite (NaOCl) followed by ethylenediaminetetraacetic acid (EDTA) for 1 min each is a standard smear layer removal protocol [20]. Various other factors such as building a pre-endodontic coronal wall before root canal debridement, [21] access cavity design, [22,23] choice of root canal irrigant [24,25], irrigant concentration, [26] usage of root canal agitation devices for final irrigant activation, [27,28] type of irrigant activation device, [29,30,31] activation protocols, [32] choice of instrument used for the root canal debridement, [33,34,35] canal curvature, and the apical root canal anatomy [36] determines the debridement efficacy and smear removal from the root canal system.

A recent report [37] has highlighted a novel automated root canal irrigation device which could be a potential alternative to current syringe needle irrigation. The claimed advantage of the automated irrigant delivery flow rates include the prevention of the operator’s fatigue and inherent irrigant extrusions. Hence, our study aimed at assessing root canal cleanliness after automated root canal irrigation using scanning electron microscopy. The null hypothesis considered in the current study was that no statistically significant difference in the debris accumulation or smear layer formation would occur with automated root canal irrigation as compared to the conventional syringe needle irrigation.

## 2. Materials and Methods

Before commencing the study, ethical approval was obtained from the institutional human ethical committee of Saveetha Dental College (Institutional Human Ethical Committee/Saveetha Dental College/Faculty/21/Endodontics/135). The research was performed as a pilot study, and the sample size was calculated with an effect size of 0.62, maintaining an alpha error of 5% and a study power of 80%. Thirty freshly extracted single-rooted human mandibular premolar teeth were obtained. These teeth had closed apices and had undergone therapeutic orthodontic extractions. Teeth chosen for the study had curvatures less than 10°. Teeth having calcifications and open apices were excluded from the study. Following tooth extraction, a curette was used to remove debris and small pieces of soft tissue was stuck to the tooth surface. To rule out the potential of numerous canals, digital radiography was used to evaluate each sample.

Following collection, the teeth were kept at +4 °C in physiological saline until the experiment. The purpose of storing at a low temperature is that it preserves the properties of the tooth and also provides potential storage medium for a longer duration. Storing the teeth in physiological saline aid prevents the growth of bacteria and dehydration. The 30 mandibular premolars’ root surfaces were dipped in a molten wax of approximately 0.2–0.3 mm thick to a depth of 1 mm apical to the cement–enamel junction (CEJ). The molten wax layer was created to mimic or replicate the alveolar bone and periodontal ligament. Once the resin was completely set, the wax was removed from the samples and embed 1 mm apical to the CEJ vertically in a self-cure acrylic. The mould cavity was filled with elastomeric impression material and the sample was then re-seated. With a no. 15 scalpel blade, the extruded material was cut to size.

With the aid of a high-speed handpiece, the access cavity was prepared using Endo Access Bur (Dentsply Maillefer, Ballaigues, Switzerland). With a fine-barbed broach, the pulp tissue was removed. A #10 stainless steel K-file (Dentsply Maillefer, Ballaigues, Switzerland), was used to negotiate the canal until the apex. The working length was determined, and the glide path was created. Based on the irrigation protocol of the study, the teeth were randomly allocated into the following into three study groups:

○ Group 1: Manual syringe needle irrigation (*n* = 10);
○ Group 2: Automated root canal irrigation (*n* = 10);
○ Group 3: Un-instrumented group (Control) (*n* = 10).

### 2.1. Group 1: Manual Syringe Needle Irrigation

All the canals were enlarged to 30 0.06 using Protaper gold rotary files. A disposable syringe with a 30-gauge side-vented needle (NaviTip, Ultradent Products, South Jordan, UT, USA) was employed for irrigation during and at the end of the instrumentation procedure. Two milliliters of 5.25% sodium hypochlorite solution (Parcan, Septodont, France) was used before starting the filing and at every change of instrument. Additionally, 2 mL of 17.5% EDTA solution (MD Cleanser, MetaBiomed, South Korea) and 2 mL of 5.25% NaOCl were alternately used. Each root canal received 2 mL of 5.25% NaOCl followed by 5 mL of a 5.25% NaOCl solution for the final irrigation. Distilled water was used as the final flush, and paper points were used to dry the canals.

### 2.2. Group 2: Automated Root Canal Irrigation

All the canals were enlarged to 30 0.06 using Protaper gold rotary files. An automated root canal irrigation device was coupled to a disposable plastic syringe with an attached 30-gauge side-vented needle (NaviTip, Ultradent Products, South Jordan, UT, USA) for irrigation in between and at the completion of the instrumentation process. The entire irrigation procedure was identical to group 1, but it was performed using an automated irrigation system [37]. Following the completion of the entire irrigation technique, the crown portion was decoronated at the cement–enamel junction (CEJ). Additionally, using a microtome LEICA SP 1600 (Wetzlar, Germany), the roots were separated bucco-lingually. The root was sputter-coated with gold and examined under scanning electron microscopy (SEM).

**Figure 1:**
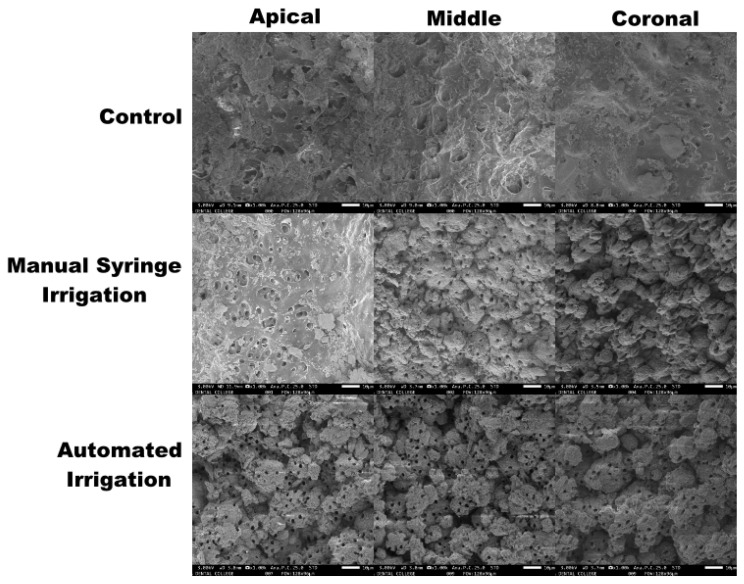
Scanning electron microscope images of the apical, middle and coronal third following irrigation of the control group, manual syringe irrigation group and automated irrigation group (1000× magnification).

Using the scoring system developed by Hülsmann et al. [38], the debris and smear layers were examined independently and scores ranging from 1 to 5 were given [39] (Table 1 and Table 2). The debris and smear layer were evaluated separately using reference images and a five score-index for each. All of the samples were scored by two unbiased evaluators (KJ and KVT) using calibration data for debris and smear layer scores. In 1000× magnification, the debris and smear layers were scored (Figure 1).

### 2.3. Statistical Analysis

Using IBM SPSS Statistics for Windows, Version 23.0, the data was examined (Armonk, NY, IBM Corp, USA). The Kruskal–Wallis test was used to determine whether there was a significant difference between the independent groups. Dunn’s pair-wise comparison test was used for an intra-group comparison.

## 3. Results

A significantly low mean debris and smear layer score (*p* < 0.05) was observed in both of study groups namely automated irrigation and control and manual syringe needle irrigation when they were individually compared with the control group. Additionally, no statistically significant difference was evident in the debris and smear layer scoring at the apical, middle or coronal region with regard to all three groups (*p* > 0.05). Nevertheless, no discernible difference between irrigation using a manual syringe needle and irrigation using an automated system was found (Table 3 and Table 4).

## 4. Discussion

The current study results showed no statistically significant differences (*p* > 0.05) between the two different irrigation modes considered. Pair-wise comparison showed significant results (*p* < 0.05) with experimental irrigation modes as compared to control, with significant differences (*p* > 0.05) with syringe needle or automated irrigation. Despite irrigant activation developments, the usage of a syringe needle irrigation system has remained the main mode of supply during root canal disinfection [40]. Recent evidence states that there are various factors and parameters involved with the syringe needle irrigation which would alter the irrigant flow and apical pressures [41].

When preparing the root canal using manual or rotary instruments, the mineralized tissues are shredded, producing a large amount of debris. A significant portion of this, which is composed of extremely fine particles of mineralized collagen matrix, is applied to the surface to create the “smear layer”.

Based on the previous periapical pressure assessment model, 1–4 mL/min is decided as an optimal irrigant flow rates to prevent the inherent apical pressures during irrigation [42,43]. However, it is impossible for an operator to maintain constant irrigant flow rates. Previous studies proved that syringe needle irrigation is difficult to standardize in the clinical scenario, as the irrigation efficiency varies based on the gender and clinical experience of the operator [44]. Hence, an automated root canal device could potentially benefit the operator by preventing the instant fatigue to operators and delivering the irrigant at constant flow rate.

To date, there is no data comparing the efficacy of the device for smear and debris removal. Hence, our study is the first one to assess smear removal and debris accumulation using automated root canal irrigation. Previous evidence clearly states the inefficiency of manual syringe needle irrigation in removal of debris and smear layer [27,28]. The current study aimed at assessing the manual syringe needle irrigation as compared to the automated root canal irrigation.

Histological evaluations of the amount of debris accumulation or residual smear layer in the root canal following instrumentation are used to measure the root canal cleanliness. The efficiency of rotary or reciprocating devices in canal cleanliness is still questionable [45,46]. Although some studies imply that reciprocating a approach leads to more debris accumulation [47], various other studies showed overall disinfection effectiveness almost comparable [45,46,48]. It has been proposed that it is the file design rather than a system’s kinematics which is responsible for better disinfection [49]. We therefore attempted to standardize the rotary file system in the current study such that there might not be any differences in debris accumulation or smear formation.

In the present study, an un-instrumented group served as a control. As far as the irrigation protocol is concerned, the proposed irrigation regimen was standardized in all the groups with no activation protocol followed. A previous study stated that the smear layer and debris removal is partly attributed to the irrigation protocol followed too [50]. As the following protocol was similar in all the groups, the effect is negligible in the current study. The irrigation protocol for the current study was selected based on the previous research report which investigated various irrigants in smear removal [51], except for the concentration of sodium hypochlorite used during irrigation. As 5.25% sodium hypochlorite has been proved to be superior to any other concentrations in terms of both efficacy and effectiveness [52,53,54,55,56,57,58], and it is the most widely preferred by world-wide endodontists [59,60], we considered evaluating using 5.25% sodium hypochlorite.

To ensure complete removal of the smear layer, the smear layer must be correctly identified. The smear layer can be identified using an electron microprobe with a scanning electron microscope (SEM) and digital image analysis. In the present study, SEM was used for analysis of the smear layer. One of the limitations of this study is that extracted, single-rooted teeth with minimal curvature were used for evaluation. Another drawback was that the irrigant activation was not followed as it could be a confounding factor since our goal was to evaluate the accumulation of debris and smear when using various irrigation techniques. Although we adhered to a standardized root canal shaping methodology in the current investigation, we did not focus on analyzing the impact of access cavity sizes and types on the evaluated outcome. Therefore, future research should focus more on evaluating molars with complex anatomy and curvatures using a standardized approach that mimics a clinical setting.

## 5. Conclusions

Within the limitation of the study, it can be concluded that manual and automated irrigation devices showed similar results for the removal of the smear and debris layer with no difference elicited between both the groups. Future studies should be performed focusing the drawbacks addressed in the present study.

## Figures and Tables

**Table 1:** Hülsmann criteria for debris scoring.

Score 1	Clean root canal wall, very slight debris.
Score 2	Slight debris.
Score 3	Moderate amount of debris,
Score 4	Substantial debris, >50% of the sample surface covered.
Score 5	Root canal sample was almost completely covered with debris.

**Table 2:** Hülsmann criteria for smear layer scoring.

Score 1	No smear layer, open dentinal tubuli
Score 2	Slight smear layer, most tubuli were open
Score 3	Homogeneous smear layer covering the major part of the surface, a few dentinal tubuli open
Score 4	Homogeneous smear layer covering the surface, no open dentinal tubuli.
Score 5	Thick nonhomogeneous smear layer covering the surface.

**Table 3:** Mean distribution of debris and smear scores among different groups.

Group	Debris Score			*p* Value	Total	Smear Score			*p* Value	Total
	Apical	Middle	Coronal			Apical	Middle	Coronal		
Manual syringe needle irrigation	1.42 ± 0.50	1.43 ± 0.53	1.22 ± 0.41	0.11	4.01 ± 0.71	1.63 ± 0.82	1.42 ± 0.53	2.21 ± 0.86	0.23	5.22 ± 0.80
Automated root canal irrigation	1.31 ± 0.81	2.12 ± 1.02	2.24 ± 0.83	0.09	5.40 ± 2.13	1.13 ± 0.53	1.32 ± 0.84	2.02 ± 0.75	0.17	5.13 ± 1.60
Control	5.01 ± 0.72	5.41 ± 0.54	5.22 ± 0.85	0.26	13.61 ± 1.54	4.82 ± 0.82	5.21 ± 0.82	5.43 ± 1.13	0.50	13.44 ± 1.12
*p* value	0.00	0.01	0.01		0.04	0.02	0.00	0.01		0.01

Note: Results are expressed in Mean ± Standard Deviation; *p* < 0.05—Significant; *p* > 0.05—non-significant.

**Table 4:** Pair-wise Comparison of Mean debris and Smear layer scores.

Study Groups	Test Statistic	*p* Value
Automated irrigation vs. Manual syringe needle irrigation	0.60	0.10
Automated irrigation vs. Control	−10.50	0.01
Manual syringe needle irrigation vs. Control	−9.90	0.01

Note: *p* < 0.05—Significant; *p* > 0.05—non-significant.

## Data Availability

Data will be made available on reasonable request from the corresponding authors.
